# Statistics of Insanity

**Published:** 1846-03

**Authors:** 


*Statistics of Insanity.*—There are 20,000 persons ascertained to be insane in England and Wales. This is, however, considerably below the actual number. They belong to every station in society; two-thirds, however, of the whole are objects of charity, and are maintained entirely at the public expense. There are 3790 private patients confined in asylums in England and Wales, out of which 1989 are males, and 1801 females. The number of paupers so confined is 7482; males 3532, females 3950; making in the aggregate of private and pauper patients, 11,272 confined in asylums in England and Wales. Out of the number above specified, 2519 are said to be "curable"—viz., 1045 private, and 1474 pauper patients. Incurable cases, 8736—males 4331, females 4405. Of epileptics there were 951, idiots 598, homicidal patients 278, suicidal cases 696. There were of the 11,272, married 3165, single 6328, widowed 1138, not known 409. Upper and middle classes 2704, agricultural1652, artisan and in-door 3868, others 2816. Criminal lunatics 257, found lunatic by inquisition 233.—*Prov. Med. and Surg. Journ., from Dr. Winslow's Edition of the Lunatics' Act.*

